# Supplementary material for: Quality Change of Citri Reticulatae Pericarpium (Pericarps of Citrus reticulata ‘Chachi’) During Storage and Its Sex-Based In Vitro Digestive Performance
Source: Foods. 2024 Nov 18;13(22):3671. doi: 10.3390/foods13223671 (PMC11594228; doi:10.3390/foods13223671)
Supplement: Supplementary file 1 [file foods-13-03671-s001.zip › foods-3217118-supplementary.pdf]

Supporting Information

Table S1. Calibration curve data for five reference compounds hesperidin, didymin, nobiletin, tangeretin, and 3,5,6,7,8,3',4'-heptamethoxyflavone (HMF) (n = 2)

Compounds	Regression equation (y = ax + b) *	R^2	Retention time (min)	Signal (nm)
Section 2.3.3				
Hesperidin	y = 6.1597 x + 19.082	0.9997	13.57	283
Didymin	y = 5.9937 x + 23.748	0.9997	14.42	283
Nobiletin	y = 14.194 x + 4.8444	0.9999	30.03	330
HMF	y = 9.0761 x - 1.4144	0.9996	34.49	330
Tangeretin	y = 14.886 x + 6.9411	0.9999	40.63	330
Section 2.4.2				
Hesperidin	y = 5.8163 x + 5.2308	0.9992	18.09	283

* y and x denoted the peak area (mAU·s) and the corresponding injection concentration (μg/ml), respectively. a and b denoted the slope and intercept of the regression line, respectively. The calibration curves exhibit good linearity (R^2>0.9990).

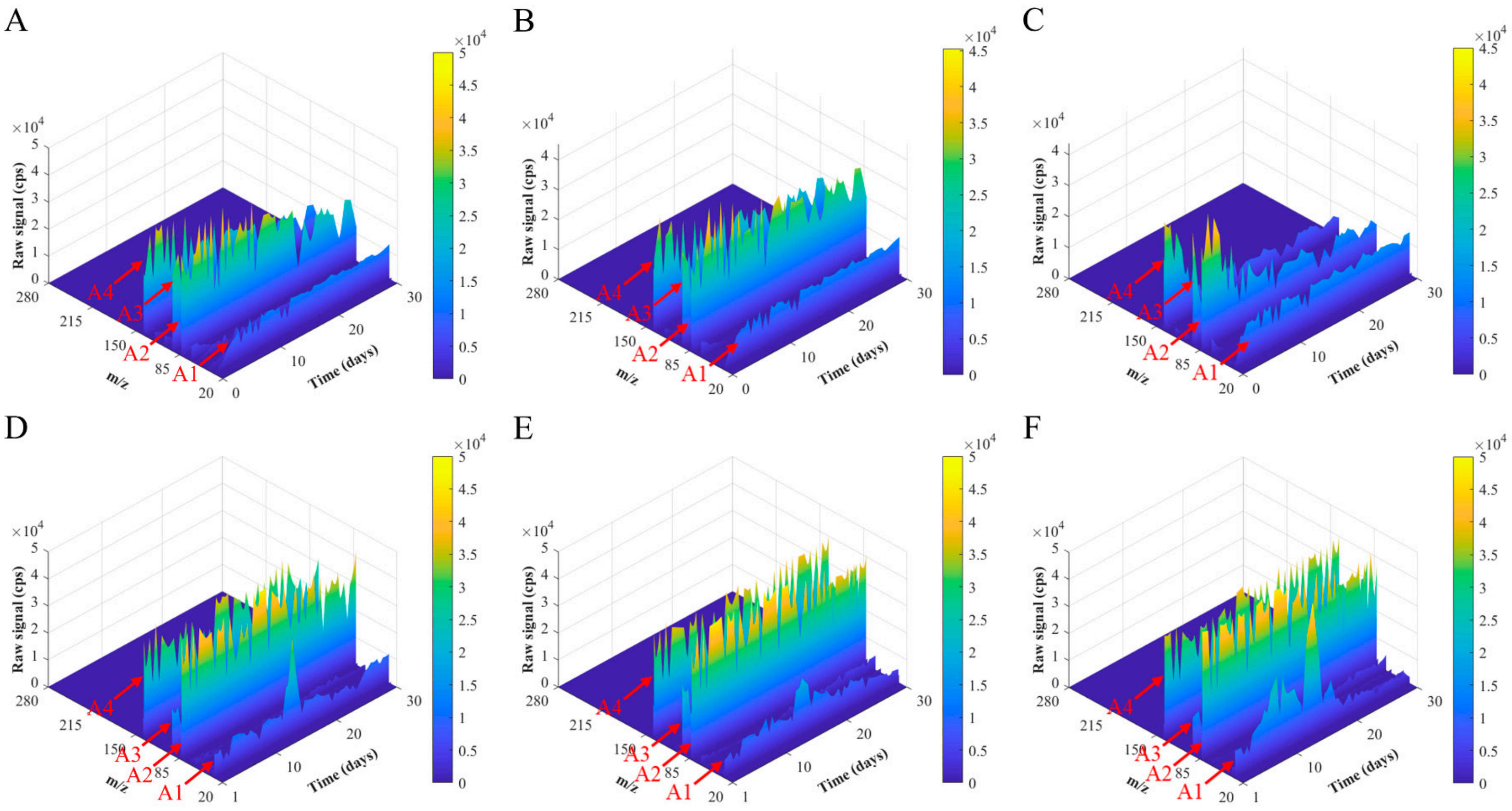

Figure S1. Three-dimensional (3D) plots of the detected spectra (*m*/*z* from 20 to 280) of the VOCs' emission from samples (A) Y2015, (B) Y2016, and (C) Y2018 during the simulated storage (25 °C and 60% RH), and from samples (D) Y2015, (E) Y2016, and (F) Y2018 during the accelerated spoilage (35 °C and 90% RH) during a month. The arrows marked the peaks of the compounds with *m*/*z* of 32, 81, 95 and 137 (compounds A1~A4 shown in Table 3).

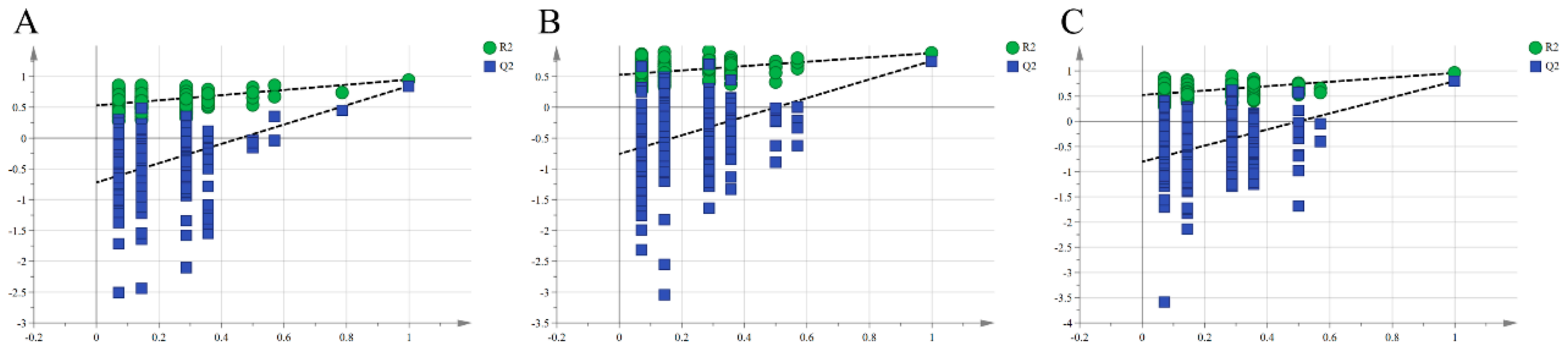

Figure S2. Performed permutations test (n = 200) with statistic parameters of [R^2 = (0.0, 0.53), Q^2 = (0.0, −0.721)] for (A) Y2015, [R^2 = (0.0, 0.528), Q^2 = (0.0, −0.757)] for (B) Y2016, and [R^2= (0.0, 0.523), Q^2 = (0.0, −0.802)] for (C) Y2018 at the first week of simulated storage in the OPLS-DA model.

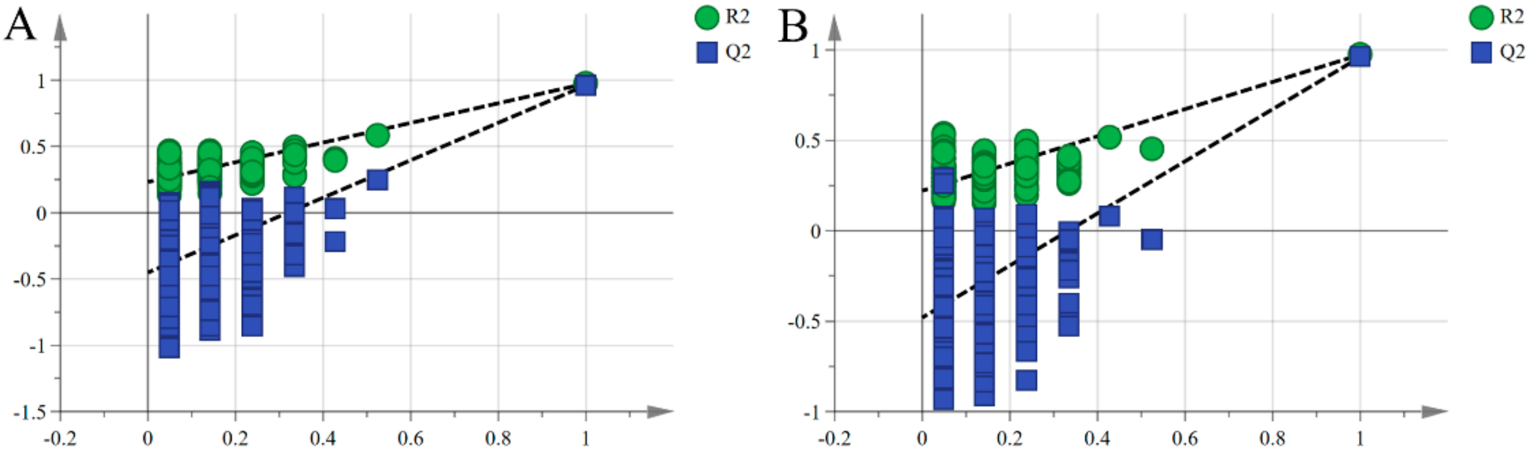

Figure S3. Performed permutations test (n = 200) with statistic parameters of [R^2 = (0.0, 0.232), Q^2 = (0.0, −0.452)] for (A) samples at the first week and [R^2 = (0.0, 0.222), Q^2 = (0.0, −0.48)] for (B) samples at the fourth week of accelerated spoilage in the OPLS-DA model.
